# Project TEAMS (Talking about Eating, Activity, and Mutual Support): a randomized controlled trial of a theory-based weight loss program for couples

**DOI:** 10.1186/s12889-017-4732-7

**Published:** 2017-09-29

**Authors:** Amy A. Gorin, Theodore A. Powers, Katelyn Gettens, Talea Cornelius, Richard Koestner, Amy R. Mobley, Linda Pescatello, Tania Huedo Medina

**Affiliations:** 1 Department of Psychological Sciences, Institute for Collaboration on Health, Intervention, and Policy, University of Connecticut, 2006 Hillside Road, Unit 1248, Storrs, CT 06269 USA; 2 Department of Psychology, University of Massachusetts Dartmouth, North Dartmouth, MA USA; 3 Department of Psychology, McGill University, Quebec, Canada; 4 Department of Nutritional Sciences, Institute for Collaboration on Health, Intervention, and Policy, University of Connecticut, Storrs, CT 06269 USA; 5 Department of Kinesiology, Institute for Collaboration on Health, Intervention, and Policy, University of Connecticut, Storrs, CT 02669 USA; 6 Department of Allied Health Sciences, Institute for Collaboration on Health, Intervention, and Policy, University of Connecticut, Storrs, CT 06269 USA; 7 Columbia University Medical Center, 622 W 168th St PH9-319, New York, NY 10032 USA

**Keywords:** Dyadic weight management, Social support, Home environment, Self-Determination Theory, Couples

## Abstract

**Background:**

Obesity risk is shared between spouses, yet existing weight loss programs focus on individuals and not the marital dyad. Given the interdependence of weight in couples, weight management outcomes might be improved by targeting joint weight loss and the creation of an interpersonal milieu that supports long-term behavior change. According to Self-Determination Theory (SDT), greater autonomous self-regulation of behaviors, and subsequently better treatment outcomes, are observed in needs supportive environments in which personally meaningful choice is supported and criticism and control are minimized. Correlational analyses confirm these pathways in weight management, with needs support from one’s spouse or partner emerging as a distinct predictor of weight loss success. Research is now needed to establish causal links and to develop and test weight loss interventions designed to facilitate the needs supportive behavior of spouses.

**Methods:**

Project TEAMS (Talking about Eating, Activity, and Mutual Support) is a randomized controlled trial testing a couples-based intervention, grounded in SDT, designed to change the social context of weight loss by training spouses to provide needs support for each other’s eating and physical activity behavior. Sixty-four couples will be randomized to either 6 months of behavioral weight loss treatment informed by SDT (SDT-WL) or to 6 months of standard behavioral weight loss treatment (BWL). Couples will attend weekly sessions for 6 months and will be assessed at 0, 3, 6, and 12 months. By bolstering needs support, SDT-WL is predicted to increase autonomous self-regulation and perceived competence and produce greater weight loss and maintenance than standard behavioral treatment. Exploratory analyses will examine the SDT process model prediction that the influence of needs support on treatment outcomes will be mediated by autonomous self-regulation and perceived competence.

**Discussion:**

This study addresses the fundamental importance of interpersonal support in weight management by focusing on couples rather than individuals and using a rich theoretical framework to train spouses in supportive behaviors.

**Trial registration:**

Clinicaltrials.gov; NCT02570009.

## Background

Spouses share obesity risk. Individuals enter into marriage with similar weight statuses, mirror each other’s weight change trajectories over time, and model dietary habits and physical activity behaviors for their children, creating a home environment that can promote or prevent obesity transmission [1–6]. Although ecological models recognize the importance of the social and interpersonal environment in the development of obesity [7, 8], weight management approaches, particularly in adults, remain focused on the individual. Behavioral weight loss treatment (BWL), the treatment of choice for adults with overweight and obesity, provides individuals with knowledge about energy balance and basic self-regulatory skills [9, 10]; however, little attention is given to the home environment and the social context in which behavioral choices are made. Spouses, despite sharing many obesogenic risk factors, are typically not involved in treatment [11, 12]. This narrow focus on the individual and the neglect of the larger interpersonal environment may contribute to the failure of current treatment programs to consistently produce long-term weight loss.

A handful of couples weight loss programs have been evaluated and, in general, have produced some additional weight management benefits [13, 14]. Most of these interventions were quite brief (8–12 weeks), had modest effect sizes, and produced diminished effects over time [13, 14]. In the most common intervention design, only one spouse in a given dyad was targeted for weight loss while the other spouse was enlisted in some manner to enhance support for behavior change [11]. In many studies there was no stated social relational framework to guide treatment nor was there a clear conceptualization of what type of involvement might be most helpful. Support was often not defined or measured, and the focus of assessment, treatment, and analysis was the index participant, not the marital dyad. In light of recent reports of the interdependence of weight and related behaviors within couples, there is a clear need to revisit couples weight loss from a theory-based lens to determine whether spouses can facilitate each other’s weight loss and to understand what type of support is most beneficial in the weight loss process.

Self-Determination Theory (SDT) offers a fresh perspective for understanding interpersonal support and motivation for health behavior change [15–17]. SDT suggests that need supportive environments, which elicit, acknowledge, and value autonomy and personal choice, establish the context for the development of self-directed, personally meaningful choice [15]. Need supportive interpersonal environments have repeatedly been associated with greater internalized autonomous self-regulation, enhanced perceived competence, higher relationship satisfaction, and greater well-being [18–21]. Autonomous self-regulation has also been consistently related to better learning, coping, and health outcomes [22–26]. These effects are most apparent for sustained change over time [26], precisely the shortfall of existing behavioral weight loss programs.

Interventions delivered in a needs supportive fashion are associated with better health outcomes [27–29], yet most studies have examined support from health care providers, not family members. Since much of weight management involves choices that are made at home [30, 31], it is imperative to examine the impact of needs support from spouses. Prior correlational research has demonstrated the importance of receiving needs support from spouses or romantic partners for dietary change [25] and for weight loss outcomes [21, 32], and that needs support operates in distinct ways from other more directive forms of support. However, the effect of needs support has not yet been tested in a randomized controlled trial on couples weight loss, and no intervention studies have attempted to train spouses to support SDT needs. A necessary next step is to develop and test an intervention designed to engender needs support within couples for weight-related behavior change.

### Study aims

The primary aim of this study is to examine the impact of a couples weight loss program rooted in SDT on weight loss outcomes and needs support, autonomous self-regulation, and competence for behavior change. This enhanced needs support condition (SDT-WL) will be compared to a more traditional model of spouse involvement (i.e., spouses attend groups but receive no training in providing autonomy support; BWL). Couples (*n* = 64) will be randomized to 6 months of either SDT-WL or BWL and will be assessed at 0, 3, 6, and 12 months.

The primary hypotheses are that compared to BWL, SDT-WL will result in: 1) greater increases in needs support from spouses, 2) greater increases in autonomous self-regulation and competence for healthy behaviors, and 3) greater weight loss at 6 and 12 months. Secondary aims will compare SDT-WL and BWL on maintenance of weight loss (6–12 months), treatment adherence, and treatment satisfaction. Exploratory analyses will compare the influence of needs support on weight loss with more directive forms of support as well as examine whether the effect of needs support on weight loss is mediated by autonomous self-regulation and perceived competence as predicted by SDT.

## Methods

### Study design (Fig. 1)

Couples will be randomized as a dyad to the enhanced needs support condition (SDT-WL) or to a more traditional model of spouse involvement (i.e., spouses attend groups but receive no training in providing autonomy support; BWL). All couples will receive 6 months of weekly weight loss group meetings and the same core information about diet and physical activity. In BWL, each member of the dyad will be encouraged to engage in healthy behaviors and weight loss efforts and to serve as a model or cue for desired behavior changes. In SDT-WL, the dyads will receive additional training in how to provide autonomy support for weight loss. Couples will be assessed at 0, 3, 6, and 12 months.

**Fig. 1:**
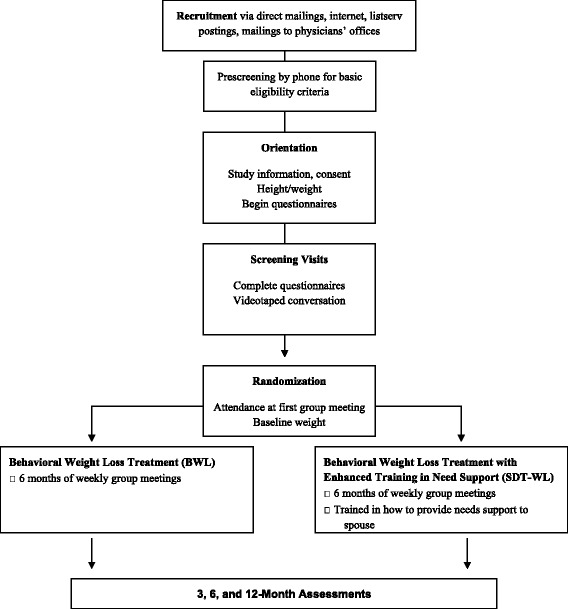
Overview of TEAMS Study

#### Participants

##### Eligibility

Eligibility will be limited to married or cohabitating couples (*n* = 64) in which each spouse is 18–70 years old with a BMI between 25 and 45 kg/m^2^. Although couples across this developmental window likely have different needs and issues, we selected this age range to be consistent with published weight loss trials [33–35]. A similar rationale was used to set the BMI criteria. Couples will be excluded if either spouse reports: current participation in a weight loss program, dieting, or taking medications that might affect weight; participation in a weight loss program in the past year; weight loss >10% of body weight during the past 6 months; current participation in any other research study that may interfere with this study; current pregnancy, lactation, < 6 months postpartum, or plans to become pregnant during the study; cancer treatment within the past year, excluding skin cancer treatment; substance abuse, dependence, average of more than 14 drinks per week, or current treatment for alcohol or substance abuse [36]; a heart condition, chest pain during periods of activity or rest, or loss of consciousness on the PAR-Q [37]; uncontrolled hypertension, history of coronary heart disease, stroke, peripheral arterial disease or having a blood pressure ≥ 160/100 mmHg as measured by study staff; chronic gastrointestinal disease; hepatitis B or C, cirrhosis, or HIV; or a significant psychiatric illness that might interfere with completion of the study. Those endorsing joint problems, prescription medication usage, or other conditions that could limit exercise or diabetes or other significant medical conditions will be required to obtain written physician consent to participate in the study.

##### Recruitment, screening, and randomization

Couples will be recruited through advertisements in the local media and screened by phone for preliminary eligibility. If eligible, couples will be invited to an in-person orientation where detailed information about the study will be provided and informed consent obtained. Once baseline assessments are completed, eligible couples will be randomized via a simple, variable-block length randomization, which ensures fairly equal allocations and will make it difficult to guess future assignments.

#### Treatment components common to both conditions

##### Treatment length

In both conditions, the 6-month treatment program will be delivered in interactive weekly group sessions (check-in period, didactic information, and group discussion) lasting approximately one hour. This meeting schedule is consistent with best practices according to recent AHA/ACC/TOS guidelines [9].

##### Interventionists

Individuals with advanced degrees in nutrition, exercise physiology, or psychology will serve as interventionists. Two Ph.D. level clinical psychologists (T.P. and A. A. G.) will train interventionists in how to work with couples, SDT, and strategies for building needs support. This ~10 h training will consist of role-playing and discussion of didactic materials and difference between conditions will be reinforced during ongoing weekly supervision.

##### Diet

Participants will be placed on a standard caloric and fat restricted diet (e.g., 1200–1800 kcals/day, ≤30% fat) consistent with published trials [33, 35, 38] and recent AHA/ACC/TOS guidelines [9]. They will receive sample meal plans, a fat and calorie guidebook and internet references, and paper copies of a daily self-monitoring diary for diet and physical activity. Participants will be allowed to record their diet in the paper diaries or use an online tracking app or program of their choice (e.g., MyFitnessPal). Interventionists will provide weekly written feedback on dietary choices.

##### Exercise

Participants will be encouraged to gradually increase their physical activity until they are engaging in 50 min of moderate intensity activity on 5 days per week (100 min/wks 1–4; 150 min/wks 5–8; 200 min/wks 9–16; 250 min/wks 17+) [9]. Brisk walking and accumulating activity through multiple short bouts will be encouraged [39], and participants will be instructed to monitor their daily exercise minutes in the self-monitoring diary or using an online tracking app or program of their choice [40].

##### Behavior therapy

Gold-standard behavioral and cognitive skills including self-monitoring, stimulus control, problem solving, goal setting, cognitive restructuring, and relapse prevention that have been used in several published weight loss trials will be taught to dyads in both conditions [9, 10]. After a 10% weight loss, keys to long-term success will be reviewed (e.g. self monitoring) and problem-solving emphasized [41].

#### Treatment components specific to the SDT condition

The primary difference between conditions is that dyads in SDT-WL will receive additional training in how to provide needs support for each other’s weight loss efforts. Adapting effective strategies from the literature on needs supportive behaviors in educators, coaches, and healthcare providers [27–29, 32, 42–45], spouses will be encouraged to: 1) elicit and acknowledge the other’s perspectives, 2) minimize efforts to control, 3) use non-judgmental, non-critical language, 4) support each other’s initiatives for change, and 5) develop empathic responding as a substitute for evaluative praise or condemnation. This type of support is distinct from more directive forms of support, such as reminding a spouse of his/her goal or encouraging certain types of eating [21]. Couples will be taught basic concepts of needs support using an “ABC” model (Table 1) and will be provided opportunities to practice these new behaviors in group meetings and receive feedback from interventionists. Spouses will be encouraged to incorporate these behaviors into their relationship between sessions and to monitor targeted support behaviors in an ongoing fashion. A variety of clinical tools will be used to facilitate adoption of these behaviors including: 1) handouts of key points in the ABC model, 2) cartoon vignette dialogues similar to those used in Faber and Mazlish’s *How To Talk To Kids So Kids Will Listen and Listen So Kids Will Talk* [46] depicting unhelpful communication patterns and more effective ways of communicating, 3) role-plays (facilitated by the interventionists) to provide couples the opportunity to practice needs supportive behaviors and receive personalized feedback from interventionists, 4) group discussions of all the concepts, vignettes and other material relevant to autonomy supportive behavior, and 5) weekly written reflection on support provided and received from one’s spouse with examples shared at group sessions.

**Table 1:** “ABCs” of Needs Support Training Objectives

Basic Concept	Training Goal	Example exercises
**A**sk to avoid assumptions	• Teach spouses to ask what would be most helpful • Teach spouses to discuss behaviors that promote weight loss and behaviors that sabotage weight loss.	• Have spouses discuss what would be most helpful; discuss what gets in the way of providing this support.
**B**e empathic	• Teach active listening skills, how to avoid destructive communication patterns, and how to validate each other’s experience.	• Have couples role play needs supportive behaviors and receive personalized feedback from interventionists.
**C**urtail control and criticism	• Teach spouses about impact of overt criticism and controlling language on behavior change efforts • Teach spouses how to identify critical and controlling styles of communication.	• Use cartoon vignettes to demonstrate dysfunctional communications and more effective patterns. • Have couples self-monitor examples of needs supportive behaviors and more controlling behaviors throughout treatment.

##### Assessments

Assessments (Table 2) will be conducted at baseline, 3, 6, and 12 months by research assistants blinded to group assignment.

**Table 2:** Data Collection Schedule for TEAMS

		Month			
Construct	Specific Measures	0	3	6	12
Demographics	Age, gender, race, ethnicity, education, income, work status, household structure, weight history, weight status of household members	x			
Anthropometrics and Behaviors	Weight and height	x	x	x	x
	Block Dietary Fat Screener	x		x	x
	Block Fruit/Vegetable/Fiber Screener	x		x	x
	Paffenbarger Activity Questionnaire	x		x	x
	Weight Control Strategies Questionnaire	x		x	x
	Self-Weighing Frequency	x	x	x	x
	Self-Report Habit Index - Automaticity	x	x	x	x
	Grocery Shopping, Meal Preparation, Shared Meal Patterns	x		x	x
	Perceived Similarity of Dyadic Eating/Exercise Habits	x		x	x
SDT Model	Important Other Climate Questionnaire	x	x	x	x
	Videotaped Conversation Coded for Types of Support	x		x	x
	Autonomous Self-Regulation-Weight Management	x	x	x	x
	Perceived Competence for Weight Management	x	x	x	x
Social Support and Relationship	Sallis Social Support Questionnaire for Eating and Exercise	x	x	x	x
	Directive support from Spouse	x	x	x	x
	Quality of Marriage Index	x	x	x	x
	Revised Dyadic Adjustment Scale (Cohesion subscale)	x	x	x	x
	Health-Related Social Control from Spouse	x	x	x	x
Process Measures	Attendance at groups		x	x	x
	Adherence to self-monitoring (completion of diaries)		x	x	x
	Health Care Climate Questionnaire			x	
	Satisfaction with program			x	

### Demographics and weight history

Basic demographic information (e.g., age, gender, race, ethnicity, education, income, work status, household composition), weight history (e.g., highest adult weight, perceived weight status), and weight status of household members will be assessed at baseline only.

### Anthropometrics

Weight will be measured in kilograms to the nearest 0.1 kg using a calibrated standard digital scale (Tanita BWB 800) with participants in light clothing and no shoes. Scale calibration will be checked periodically with known weights. Standing height will be measured in participants without shoes using a wall-mounted Harpenden stadiometer. All anthropometric measures will be taken in duplicate and the mean will be used in analysis.

### Weight-related behaviors

#### Diet

The Block Dietary Fat Screener, a brief 17-item screening tool, will be analyzed using existing prediction equations to generate point estimates of total fat, saturated fat, percent calories from fat, and cholesterol (http://NutritionQuest.com). The 7-item Block Fruit/Vegetable/Fiber Screener will be analyzed using existing prediction equations to generate point estimates of total fruit/vegetable servings, Vitamin C, magnesium, potassium, and dietary fiber (http://NutritionQuest.com).

#### Physical activity

The Paffenbarger Activity Questionnaire (PAQ) [47]; a measure with high test-retest reliability [47] that has been shown to be correlated with weight control and cardiovascular fitness measures [48], will assess the number of hours per day an individual spends doing various levels of physical activity or inactivity in a typical weekday and typical weekend day. Participants will also complete the 4-item Self-Report Habit Index (Automaticity Index) [49] in respect to exercise and report the location and time of day they most often exercise to capture habitual behavioral patterns. Family patterns around exercise times and similarity with spouse’s exercise habits will be assessed with items constructed for this study.

#### Weight-related strategies and habits

The Weight Control Strategies Scale, a 30-item measure that is sensitive to change during a behavioral weight loss program, predictive of weight loss success, and associated with expected energy balance behaviors [50], will assess the use of specific weight management behaviors (e.g., chose low-calorie options; kept a record of the type and amount of food I ate) in the past 6 months. Additional questions will be added to capture use of online and mobile fitness apps and websites and whether individuals participate in any commercial weight loss programs during the study period. Frequency of self-weighing during the past month will be assessed using a 1-item measure that has been widely used in weight management research [51, 52]; participants will also report the location and time of day of weighing via questions from the Self-Report Habit Index (Automaticity Index) [49]. Grocery shopping practices, patterns of shared family meals, and similarity with spouse’s eating habits will be assessed with items constructed for this study.

### Self-Determination Theory measures

Needs support will be measured in two ways. First, participants will complete items adapted from the Important Other Climate Questionnaire (IOCQ) [53]. The measure assesses the perceptions of needs support that partners experience from one another (e.g., “My partner conveys confidence in my ability to control my own weight”). Spouses will also report on their own needs supportive behavior by completing a 14-item scale similar to the IOCQ, but written in the first person (e.g., “I have conveyed confidence in my partner’s ability to control his/her own weight”). Items assessing more controlling or directive forms of support will be added to both versions of the IOCQ consistent with the published literature [32]. As an objective measure of needs support, couples will be videotaped in a structured 10-min conversation prior to the intervention and again at the 6 and 12 month visit. Pairs will be asked to discuss a scenario related to the their weight management efforts (e.g. “You get home from work and see that your spouse is watching TV. He/she is supposed to be at the gym. What would you say?”). These interactions will be coded for specific needs supportive (e.g., acknowledging feelings, asking what the partners wants) and controlling behaviors (e.g., criticizing, “should” statements) by two independent observers and inter-rater reliability will be calculated.

Autonomous self-regulation will be asssesed with a 12-item Reasons for Weight Control scale adapted from Lesveque et al. [25] which asks participants to report their reasons for losing weight. Half of the items reflect autonomous motivation (e.g., “Because I feel that I want to take responsibility for my own health”) and half reflect controlled motivation (e.g., “Because I would feel guilty or ashamed of myself if I did not try to control my weight”).

Participants will also complete the 4-item Perceived Competence Scale (PCS) used extensively by Williams and colleagues in related health domains (e.g., diabetes management) [28, 42] to assess competence for weight management.

### Support and relationship quality measures

The Sallis Support Scales [54] will examine more directive forms of spouse support for healthy eating and exercise. The scales have adequate reliability and validity, show some associations with exercise and eating behaviors, and have been utilized in previous weight loss studies [21, 55]. Spouses will also report on their own supportive behavior by completing a scale similar to the Sallis Support Scales, but written in the first person (e.g., “I reminded my partner not to eat high fat, high salt foods”).

#### Relationship quality

To assess marital satisfaction and cohesion, participants will complete 6-items from the Quality Marriage Index (QMI) [56] and 4-items from the Revised Dyadic Adjustment Scale [57]. Both scales have been used extensively and have adequate psychometric properties. Health-related social control will be assessed using 8-items from Tucker [58]. Indirect control will be assessed with 4-items (e.g., My spouse/partner expects me to try to stay healthy) and behavioral response to social control will be assessed with 4-items (e.g., Hide the behavior from my spouse).

#### Measures of adherence

Data will be collected on the number of intervention visits attended. Attendance at treatment sessions is anticipated to decline over time, but, in keeping with prior studies in the weight control literature, those who attend more sessions are expected to have better weight loss outcomes [59, 60]. Adherence to self-monitoring, a known predictor of treatment success [61, 62], will be assessed by tracking the total number of self-monitoring diaries that are completed and turned into the group leader during the interventions.

#### Perceived impact of intervention

A measure will be created for the study to assess participants’ perceptions of the effects of the training on their attitudes and behaviors. Items will include free response questions and forced-choice items and will be completed at the 6 month assessment.

##### Quality control and safety procedures

Intervention manuals specific to each condition will be developed. All group sessions will be audiotaped. To assess treatment fidelity, approximately 20% of sessions will be reviewed by study investigators to: 1) determine whether they can identify which condition the particular session belongs to, 2) assess whether key elements in each session are covered, and 3) whether any cross-contamination occurred. A data safety monitoring board (DSMB) will be established to assure safety and study integrity. Two faculty members with expertise in health behavior change and biostatistics will meet every 6 months to review progress and adverse events. They will make recommendations to the PI and report any concerns to the Institutional Review Board or Office of Research Administration.

##### Procedures to retain the sample

Retention at follow-up visits will be promoted through strategies used effectively in other studies (e.g., collecting contact information of relatives and friends who can be contacted if unable to reach the participant). For each data collection visit, participants will be scheduled by phone or email, sent reminders via mail/text/or email, and called/texted/or emailed the day before. Missed visits will be rescheduled. Childcare and costs for transportation will be provided to participants if these are significant barriers to assessment visits. If necessary, assessments will be completed at participants’ homes, workplaces, or an agreed upon public location. Participants may also be offered the option of completing assessments by phone or online. Participants will be given an honorarium of $10 at 3 months, $25 at 6 months, and $40 at 12 months for completing assessments. We expect completion rates ~90% at follow-up.

### Statistical analysis

#### Sample size, power and effect size

We will have 80% power for a two-tailed test at alpha = .05 to detect an effect size equal to d = .71 at the dyad level, and .62 at the individual level (adjusting for interdependence within dyads) [63] for our primary weight loss comparison. Although our statistical power is limited, we will be able to calculate the 95% confidence interval around the point estimate of the SDT-WL intervention effect that will allow us to conduct an accurate power analysis for a future efficacy trial.

#### Preliminary analyses

Baseline characteristics in SDT-WL versus BWL will be examined for comparability on demographic, weight history, psychosocial, and behavioral measures using multilevel regression models to account for interdependence within dyads (i.e., the tendency for couple members to be more similar to each other than to other participants) [63]. The distributional properties of any continuously scaled variables will be examined to determine if normalizing transformations should be applied. If group differences are noted on any baseline variables they will be statistically controlled by employing such variables as covariates in outcome analyses.

#### Statistical analyses to support the primary aims

Structural regression models that treat couple members as nested within dyads will be used to examine primary aims of the study [63] using the R package lavaan [64]. To analyze the primary hypothesis that SDT-WL versus BWL will achieve greater increases in perceived needs support from baseline to 6 and 12 months, needs support at each time point will be used as an outcome variable, controlling for baseline needs support. Full information maximum likelihood estimation (FIML) will be used to account for missing data. Any relevant between- and within-dyad covariates based on the preliminary analyses described above will also be included in the analysis. Dyadic analysis will examine primary hypotheses that SDT-WL versus BWL will display greater autonomous self-regulation and competence for healthy behaviors (i.e., eating and physical activity) and greater weight loss at 6 and 12 months using the same strategy outlined above.

#### Statistical analyses to support the secondary aims

The same dyadic data analysis procedure as noted above will be employed to examine weight loss maintenance from 6 to 12 months, treatment adherence, and treatment satisfaction between groups. This strategy will also be used to explore the effect of needs support on weight change from baseline to 6 and 12 months, controlling for the effects of other, more directive forms of support. Finally, mediation analysis will explore SDT variables (i.e., autonomous self-regulation and competence for healthy behavior) as mediators of the effect of perceived needs support on weight outcomes.

## Discussion

Married or cohabitating couples are an important dyad to target for joint behavior change and weight loss. Most eating and activity decisions are made in the home [30, 31], therefore it makes intuitive sense to involve other adults from the home in treatment to facilitate healthy behavior change. Converging evidence also suggests that weight is interdependent within dyads – spouses are sensitive to both weight gain and weight loss in their partners, often mirroring changes in either direction themselves [2, 5, 6]. While social factors clearly influence weight and related behaviors, our understanding of how to involve spouses in the weight loss process is in its infancy [11]. To date, most couples weight loss programs have been of limited duration, lack a social relational model to guide treatment development and assessment, and have had minimal impact. Self-Determination Theory (SDT) is a theory of motivation that proposes three psychological needs that are essential to health and well-being – relatedness, competence, and autonomy [15–17] and provides a framework for working with couples to support behavior change. The current study tests a SDT-based couples weight loss program that teaches spouses how to support each others needs and create an interpersonal environment that promotes sustained behavior change.

The study is innovative in several ways. Married couples, not individuals, will be the unit of treatment and analysis. The intervention will be delivered to both spouses and the statistical approach will fully utilize the dyadic nature of the data to provide a nuanced understanding of how couples lose weight together. In contrast to earlier couples interventions [11, 13, 14], a clear social relational framework will guide both the treatment and assessment protocols. Needs support is well defined and is distinguished from other types of support (i.e., directive support) both in how couples are taught to work together to accomplish their goals and in how support is measured in the assessment battery. Several types of support will measured subjectively (self-reports) and objectively (videotaped observations) over the 12-month study allowing for a prospective test of the SDT model and an analysis of the unique contribution of autonomy support to weight loss outcomes.

Project TEAMS provides an important first test of the benefits of approaching weight management as a couples health issue. If couples can achieve weight loss together, the whole family might benefit. Children of parents living with obesity are 12 times more likely to have obesity than children with normal weight parents [65]. Moving the focus of study from the individual to the married couple has the potential to improve weight loss outcomes and promote a healthier weight status in all family members.
